# The Institutional Use of Alcoholic Liquors

**Published:** 1912-06-29

**Authors:** 


The Hospital

The Workers' Newspaper of Administrative Medicine and Institutional Life, Administration, National Insurance and Health.

No. 1356, Vol. LII. SATURDAY, JUNE 29, 1912. PRICE ONE PENNY.

### THE INSTITUTIONAL USE OF ALCOHOLIC LIQUORS.

The consumption of alcoholic liquors, it has often been pointed out, has immensely decreased of late years in hospitals. Indeed it has been asserted that one of the most remarkable changes in hospital administration during the past twenty or thirty years has been the ousting of alcohol from the position of popularity which it occupied before that time. Whenever a hospital worker who has had experience of institutional life during the middle of the last century, or even the latter end of it, records his recollections, he lays stress, invariably, on this: namely, that the bill for alcoholic liquors has been diminished by 80 per cent. The fact is undoubted; the interpretation of it which is often given is open to argument. There can be little question that there has been a marked increase in the soberness of the community as evidenced by the statistics of inebriety. Whether this fact has any connection with the decline in the consumption of spirituous liquors in hospitals—whether or not, in other words, the commonly accepted explanation of this decline, based on the assumption that institutional temperance is on the increase, is true, and finally whether or not there is any reason to believe that the inmates of hospitals are more sober nowadays than their predecessors were fifty years ago—these are points which, however interesting they may be from an academic point of view, are scarcely pertinent to the main theme of interest to hospital workers in connection with this question of alcohol.

This main theme is whether the neglect of alcoholic liquors in favour of the so-called temperance drinks, and of the more expensive synthetic and chemical stimulants now so largely employed, is justified by results. This inquiry is of some importance to every hospital worker. Good alcohol, notwithstanding the attacks which are made upon it from time to time, both in the profession and outside it, still stands, by the agreement of the majority of authorities, as one of the most useful adjuvants which the medical man has at hand in certain cases. No one attempts to defend the abuse of alcohol, either at the table or at the bedside; but it is folly to maintain, in the face of all experience to the contrary, that the discriminate use of alcoholic liquors, in health or in sickness, is inimical to the interests of the user. It has recently been proved that the ingestion of a certain amount of wine with an ordinary meal is a distinct aid to digestion—a fact which has been known from very ancient times. It still remains true, as most physicians will readily admit, that alcohol, in certain cases of disease, is one of the stand-bys which we cannot afford to eliminate from the pharmacopœia, however much we may deprecate the abuse of this highly valuable drug.

That being the case, it is worth while to inquire whether hospitals are justified in curtailing the use of alcoholic liquors and in discontinuing the old custom which allowed certain patients a glass of beer with their meals. It is easy to understand the value of a good glass of beer to a convalescent patient: both from the dietetic and from the psychical point of view the addition of this drink to the dietary of patients who can safely be allowed to use it is to be commended. It is very much better than allowing the patients to drink aerated waters flavoured with fruit syrups or the generally indifferently made cocoa or tea which is served out in most wards. Measured by calories—necessarily a fallacious method of estimating the value of a given diet—the addition of beer to a patient's dietary may not materially increase the food value of the diet, but it is now well known that the extractives of beer have in themselves an appreciable food value, whereas the importance of beer as a condiment is admitted by all except those who are fanatically opposed to alcoholic liquors in any form. Obviously the answer to this plea for the admission of beer to the diet scale of the average hospital patient is that such an innovation—which is however not an innovation but merely a return to an old practice which has fallen into disuse—cannot be sanctioned because of the expense entailed in carrying it out. A moderate consumption of beer, especially when the liquor is obtained, as in the German hospitals, direct from the manufacturers at contract prices, will not largely increase the annual expenses, and it is possible that the value of the addition will fully justify the trial. While we do not urge the wholesale renewal of the old custom, we think there is at all events sufficient evidence in its favour to make hospital authorities consider the advisability of removing the now general embargo on beer in hospital wards.

When we come to the question of wine and spirits it is evident at once that in many cases the boasted saving on the alcohol bill is more than compensated by the increase in the drug bill owing to the indiscriminate use of costly and fashionable synthetic and proprietary stimulants, animal extracts, and new pharmacopœial preparations which are supposed to be "ever so much better than alcohol." Whether they merit this claim or not is a matter that need not be here discussed. The fact remains that their superiority to the older, well-tried stimulant, alcohol, is not so incontestable as to warrant their greater expense and their almost universal employment in hospitals. This tendency to reject the older stimulant in favour of the newer extractives is responsible on the one hand for the diminution of the consumption of alcohol in the wards, and on the other for the increase in the bill for other stimulants. A return to the older methods may not be without benefit not only to the patients but to the institution as well. We hold no brief for the brewer or the wine merchant, and our interest lies solely in the institutional and medical aspect of the question we have touched upon. The views we have expressed may possibly seem reactionary to some of our readers who are unacquainted with the newer researches upon the subject of the food value of alcohol; but we trust at least that they merit impartial consideration.

